# Prevention of mother-to-child HIV transmission in resource-limited settings: assessment of 99 Viramune Donation Programmes in 34 countries, 2000–2011

**DOI:** 10.1186/1471-2458-13-470

**Published:** 2013-05-14

**Authors:** Joël Ladner, Marie-Hélène Besson, Mariana Rodrigues, Kelley Sams, Etienne Audureau, Joseph Saba

**Affiliations:** 1 Rouen University Hospital, Epidemiology and Public Health Department, Rouen University Hospital, Hôpital Charles Nicolle. 1, rue de Germont, Rouen cedex, 76 031, France; 2 Axios International, 7 boulevard de la Madeleine, Paris, 75001, France; 3 Biostatistics and Epidemiology Unit, Hôtel Dieu, Assistance Publique Hôpitaux de Paris, Paris Descartes University, Paris, France

**Keywords:** Anti-retroviral agents, Delivery of health care, HIV infection, Infectious disease transmission, Vertical, Models, Organizational, Nevirapine

## Abstract

**Background:**

Transmission of HIV from mother-to-child during pregnancy, labor, or breastfeeding is the primary cause of pediatric HIV infection in sub-Saharan Africa. A regimen of single-dose nevirapine administered to both HIV-positive pregnant women and their infants has been shown to lower the risk of mother-to-child transmission (MTCT) of HIV. In an effort to facilitate scale-up of PMTCT programs in low-income countries, Boehringer Ingelheim, the manufacturer of Viramune (branded nevirapine), initiated the Viramune Donation Programme (VDP) in 2000. The aim of this study was to evaluate the impact of the VDP on participating institutions.

**Methods:**

A total of 164 institutions in 60 countries were included in the VDP over its 11-year duration. An online quantitative and qualitative questionnaire was submitted to all program managers. The questionnaire collected data on the impact of the VDP on initiation and scale-up of PMTCT services, operational capacity, national PMTCT policies, access to funding, and national and international partnerships. Participants were asked for their opinion of how VDP was perceived by different stakeholders (medical community, patients, government authorities, communities).

**Results:**

Ninety-nine managers (60.4%) in 34 countries responded to the online questionnaire; 89 of institutions (89.9%) were located in Africa The most positive aspects of the VDP identified were: helped to expand PMTCT services (85.9% of program managers), reduced stigma against HIV-positive pregnant women, increased social support mechanisms (78.8%), fostered partnerships with national and international organizations (69.0%), and encouraged access to donor funding (63.0%). Implementation of the VDP triggered improvements in training hospitals and logistical capacity and was associated with changes in policy strategies at the national level.

**Conclusion:**

A drug donation program such as the VDP can act as a catalyst for systemic changes at the institutional and national levels. The VDP provides a model for how private initiatives can have a significant impact on public health issues and foster diverse public-private partnerships among governments, commercial organizations, local institutions, and international NGOs.

## Background

It is estimated that two million children in the world were living with HIV in 2011, and 370,000 were infected during the same year, mainly through mother-to-child transmission (UNAIDS, 2011). Transmission of HIV from mother-to-child during pregnancy, labor, or breastfeeding is the primary cause of pediatric HIV infection in sub-Saharan Africa, resulting in approximately 370,000 new HIV infections in 2009 [1-4]. However, over the last 10 years, mother-to-child transmission of HIV has been nearly eradicated in developing countries [5] following directed efforts by both public and private entities.

In 1994, a study by Conner et al. demonstrated that zidovudine (ZDV) administered to HIV-positive pregnant women ante and intra partum and to neonates for six weeks after birth reduced the risk of mother-to-child transmission (MTCT) of HIV by about two-thirds in non-breastfeeding women [6]. In 1999, the HIVNET 012 study showed that a single dose of nevirapine administered to HIV-positive pregnant women at the onset of labor and a single dose administered to their infants within 72 hours of birth lowered the risk of MTCT of HIV by 47% in a breastfeeding population [7]. These results demonstrated the efficacy of a regimen for prevention of MTCT (PMTCT) of HIV that was feasible in low-income countries and provided an opportunity to narrow the PMTCT gap between low- and high-income countries. The international community had long been waiting for such positive results, and the proof-of-concept data generated a significant momentum to make the intervention available in developing countries.

In 2001, the World Health Organization recommended a short course of anti-retroviral (ARV) therapy in late pregnancy or during labor as part of PMTCT programs, in addition to safer delivery practices and guidance on breastfeeding [8,9]. The recommended regimens included zidovudine (ZDV) alone or in combination with lamivudine (3TC) or single-dose NVP. Of these regimens, single-dose NVP administered to the mother during labor and to the child after birth was the simplest, in part because mothers could take their oral dose of NVP at home once labor had started [10]. Early provision of single-dose NVP, often at the time that pregnant women tested positive for HIV, has helped to increase the number of such women who received this intervention [11]. A recent study emphasized the importance of increasing PMTCT access in reducing maternal and infant mortality and transmission of HIV to infants [12].

In response to the momentum generated by the results of HIVNET 012 and in an effort to facilitate scale-up of PMTCT services in low-income countries, Boehringer Ingelheim, the manufacturer of Viramune (branded Nevirapine) initiated the Viramune Donation Programme (VDP) in 2000. In 2006, the WHO revised its PMTCT guidelines in response to the availability of new data and new treatment regimens [13,14]. The guidelines replaced single-dose NVP with longer courses of ARV therapy administered to both the mother during pregnancy and the child after birth [14].

A number of studies have assessed a variety of approaches to implementing PMTCT programs in low-resource countries. For example, an analysis of PMTCT programs supported by the Elizabeth Glaser Pediatric AIDS Foundation in sub-Saharan Africa demonstrated that HIV counseling, testing, and delivery of PMTCT services is feasible in low-resource countries [11,15]. However, the availability of antenatal care services varies depending on the economic resources of individual countries and is dependent on human and logistic resource capacity, as well as health care workers [16]. In addition, lessons learned with respect to programs that expand access to drugs could be very useful and serve as a model for the management of other diseases, including chronic health diseases such as cancer, diabetes, and cardiovascular diseases.

The 11-year history of the VDP provided an opportunity to better understand the long-term effects that result from the availability of a new health intervention in resource-limited countries. The aim of this study was to evaluate the role that the VDP has played in the initiation and scale-up of PMTCT services in developing countries.

## Methods

Axios International has been responsible for managing, monitoring, and distributing NVP for the VDP. To facilitate the implementation and management of the programme, Boehringer Ingelheim contracted the services of Axios International to implement, manage, and monitor the distribution of NVP. Participation in VDP is based on expressed interest by local governments, charitable organizations, NGOs and other health care providers. Before a donation is received, local government approval to receive the drug must be sought. Between 2000 and 2011, the VDP provided 2.3 million mother-child pairs of NVP at no cost to low-and middle-income countries worldwide. In the VDP, nevirapine (NVP) was provided free of charge to low-income countries for use in PMTCT. The donation was available to governments (Ministry of Health, MoH), non-governmental organizations (NGOs), and international agencies, with the approval of national health authorities. On a regular basis, each institution included in the VDP submitted a progress report documenting activities conducted for the period covered and requests for additional doses based on their progress.

First, a questionnaire was developed, pilot-tested by phone, and validated with 20 randomized institutions included in the VDP. The final questionnaire was administered online to all institutions that participated in the VDP over its 11-year history. All VDP institutions received a link to the questionnaire by email and hard copies were also made available on request. The online standardized questionnaire was available in English and French and included both structured, closed questions and open-ended questions. The questionnaires were completed by the institutional VDP Program managers.

Non-responding institutions received four follow-up emails and attempts were also made to contact these institutions by phone. The study was reviewed by the Ethics Committee (Comité de Protection des Personnes Nord Ouest 1 in France), which determined that a statement of Ethics Committee was not necessary.

This study used a mixed-method approach to provide a descriptive impact evaluation from the perspective of participating VDP institutions, enabling the presentation of both quantitative measurements and qualitative insights. Qualitative data were systematically collected and analyzed from each participant in the form of online verbatim and transcripts.

The impact of the VDP was assessed in the following areas: initiation and scale-up of PMTCT services, operational capacity, change in national PMTCT policies, initiatives and related Ministry of Health budgets, access to funding, national and international partnerships, health worker training, and health worker attitudes toward HIV-positive pregnant women. Program mangers were also asked for their opinions on how the VDP was perceived by the medical community, patients, government authorities, and members of the local community with access to VDP sites. A 6-point Lickert scale (from very positive impact to very negative impact, including “don’t know”) was used to measure responses among the four stakeholder groups.

The impact of the VDP was also assessed in the following areas: stigma reduction, level of social support for seropositive women, patient access to PMTCT services, drug supply issues, benefits and challenges of donation programs, and other aspects HIV/AIDS prevention and treatment.

To analyze the range of experiences and points of view reported, the quantitative and qualitative data were extracted and indexed to generate the following analytical categories: access to care, operational impact, impact on funding, and perception of VDP among key constituencies. Analyses were descriptive, with qualitative variables expressed in percentages and quantitative variables presented as mean values with their standard deviation (SD), median (M), and interquartile range (IQR).

Role of funding source. Boehringer Ingelheim had no role in study design, data collection, data analysis, data interpretation, or writing of the paper. The authors had full access to all data in the study and had final responsibility for the decision to submit for publication. Although Axios International received financial compensation for managing the VDP, the results presented here are a complete and accurate representation of the data collected through the survey tool. As such, the data describe the experiences and perceptions of personnel participating in the VDP, rather than the views of Axios or Boehringer Ingelheim.

## Results

A total of 164 institutions in 60 countries were included in the VDP over its 11-year duration. Of the 164 beneficiary institutions to which the online questionnaire was sent, program managers at 99 institutions (60.4%) in 34 countries responded and were included in the analysis (Table 1). Of these 99 institutions, 35 were Ministries of Health (35.5%), 53 were NGOs (53.4%) and 11 were international agencies (11.1%) (Table 1). Sixty of the responding institutions (60.6%) implemented the VDP between 2001 and 2004, with a mean follow-up of 3.1 years (SD=2.8, M=3.0, IQR=1.1-2.1) among all 99 institutions. E-mails bounced back from 49 institutions (29.9%) and these institutions were deemed unreachable. Of these 49 institutions, 24 programs (50.0%) were managed by Ministries of Health, 23 by NGOs (47.9%) and one by an international agency (2.0%). 24 of these 49 institutions (50.0%) implemented the VDP between 2001 and 2004, with a mean follow-up of 2.2 years (SD=2.1, M=2.0, IQR=0.9-3.1). 16 other institutions (9.7%) did not respond, although the emails were not returned. Of these 16 institutions, 10 were Ministries of Health (62.5%) and 6 were NGOs (37.5%); 13 of these 16 institutions (81.2%) implemented the VDP between 2001 and 2004, with a mean follow-up of 2.6 years (SD=2.8, M=2.5, IQR=0.8-3.2).

In this study, 89 of the 99 responding institutions (89.9%) were located in Africa (Table 1). This is consistent with the higher HIV prevalence rates in African countries, which made PMTCT a higher priority and increased the value of testing pregnant women for HIV. A separate analysis of results from the 89 African respondents only was similar to those for all 99 respondents (data not shown).

The positive aspects of the VDP identified by the 99 participating institutions were: helped to expand PMTCT services (reported by 85.9% of the VDP Program managers), reduced stigma against HIV-positive pregnant women and increased social support mechanisms (declared by 78.8% of the VDP Program managers), fostered partnerships with national and international organizations (69.0%), simplicity of the program (67.7%), encouraged access to donor funding (63%), helped to start PMTCT services (54.5%), helped to build a logistics infrastructure (42.4%), and increased training capacity (40.4%). The most important contributions of the VDP reported by program managers were an increased number of PMTCT facilities (reported by 77.8% of program managers), an increase in the quality of PMTCT services provided (56.6%), and an impact on national PMTCT policies and strategies (53.5%).

### Access to care

Improved access to PMTCT services was reported by 84.8% of program managers. With respect to the impact of the VDP on the different components of comprehensive PMTCT services, 83.8% of respondents indicated that the VDP increased the number of women receiving VCT, 60.6% reported an increase in the number of women receiving highly-active antiretroviral therapy (HAART), and 47.5% said there was an increase in the number of women receiving antenatal care. The VDP did not appear to impact the number of men receiving VCT.

Open-ended responses indicated that the VDP helped speed the pace of implementing PMTCT, enabled implementation of anti-HIV efforts by providing screening and access to ARVs, and allowed government agencies to extend their activities by relieving part of their PMCTC burden. Open-ended responses also indicated that the improvement and scale-up of PMTCT services that resulted from participation in VDP increased patient access to such programs. Factors related to the VDP that increased patient access included: increase in the number of PMTCT sites; increase in the number of women seen in ANC visits, undergoing testing, receiving NVP and undergoing treatment with ARVs; adoption of PMTCT services; and health worker attitudes.

### Operational impact

Table 2 shows the impact of the VDP in several key operational areas. Program managers reported that the VDP had a positive impact on training capacity and skills development among health workers, with 90% of respondents reporting a high or very high impact. 72% of program managers also reported that the VDP had a high or very high positive impact with respect to professional changes in health workers. These changes include an increase in the number of workers trained, the development of integrated guidelines used for staff training, and consistent engagement of health workers in providing PMTCT services, which led to improved skills over time.

**Table 1:** Countries and number and type of institutions included in the study

**Region**	**Country**	**Number of institutions included**	**MoH**	**Type NGOs**	**IA**
Africa	Benin	2	1	1	0
	Burkina Faso	5	2	2	1
	Cameroon	5	0	4	0
	Chad	2	0	2	0
	Cote d’Ivoire	3	3	0	0
	DRC	5	0	5	0
	Ethiopia	2	0	2	0
	Gabon	1	0	1	0
	Ghana	1	0	1	0
	Guinea Bissau	1	0	1	0
	Guinea Conakry	1	1	0	0
	Kenya	10	3	5	2
	Lesotho	1	1	0	0
	Liberia	1	1	0	0
	Malawi	7	1	1	5
	Mali	1	1	0	0
	Mozambique	4	1	3	0
	Niger	1	1	0	0
	Nigeria	7	2	5	0
	Republic of Congo	2	1	1	0
	Rwanda	1	1	0	0
	Senegal	1	1	0	0
	Sierra Leone	1	1	0	0
	South Africa	5	2	3	0
	Swaziland	1	1	0	0
	Tanzania	9	1	6	2
	Togo	2	1	1	0
	Uganda	2	1	0	1
	Zambia	3	0	2	0
	Zimbabwe	3	2	1	0
	*Sub total*	*90*	*31*	*48*	*11*
Asia	Cambodia	1	1	0	0
	Nepal	1	0	1	0
	Vietnam	1	1	0	0
	*Sub total*	*3*	*2*	*1*	*0*
Caribbean	Dominican Republic	1	1	0	0
	Haïti	4	0	4	0
	*Sub total*	*5*	*1*	*4*	*0*
Europe	Ukraine	1	1	0	0
Total		99	35	53	11

Viramune Donation Programme, 2000–2011 (N=99).

DRC : Democratic Republic of Congo. MoH: Ministry of Health. NGOs: Non-Governmental Organisation. IA: International Agency.

**Table 2:** Impact of Viramune Donation Programme on PMTCT national and institutional budgets, fostering partnerships, and health workers abilities, 2000–2011 (N=99) (results are expressed in percentages)

	**Very high impact**	**High impact**	**No impact**	**Negative impact**	**Very negative impact**	**Don’t know**
On the MoH budget for PMTCT over the past 10 years	22.2	41.4	12.1	0	1.0	23.3
On contribution on budget changes at national level	17.2	33.3	13.1	1.0	0	35.4
On help for access to donor funding at institutional level	33.0	29.9	25.8	0	0	11.3
On fostering national and international partnerships	23.5	45.9	13.3	1.0	0	16.3
On training and skills development of health care workers	33.3	56.6	6.1	0	0	4.0
On professional changes in health workers	25.3	46.5	18.2	0	0	10.1

MoH: Ministry of Health.

### Impact on funding

The VDP also appeared to have impacted several aspects of funding (Table 2). The program managers attributed increased funding at the institutional level to increased demand for services created by the VDP and increased credibility of institutions involved with the VDP due to the success of their programs. In addition to free NVP donated through VDP, 90.0% of institutions reported receiving outside funding from a variety of donors to support their PMTCT programs. Half of the responding institutions (53.2%) received funding from the US government.

### Perception of the VDP among Key constituencies

Qualitative analysis was also undertaken with respect to program managers’ perceptions of how the VDP was received by four key constituencies: the medical community, patients, government representatives, and the local community (Table 3). Key themes related to the positive perception of the VDP in the medical community were: gave physicians the opportunity to offer patients an effective strategy for PMCTC and to strengthen facilities, capacity and services at their institutions; initiated/facilitated better involvement of the medical community in PMTCT; and provided resources that helped to fill gaps in low-resource settings.

**Table 3:** Perception of Viramune Donation Programme (VDP) by the medical community, patients, government authorities, and community, 2000–2011 (N=99) (results are expressed in percentages)

	**Very well**	**Well**	**Neutral**	**Badly**	**Very badly**	**Don’t know**
Medical community	73.8	20.2	3.0	0	0	3.0
Patients	52.5	30.3	7.1	0	0	10.1
Governments authorities	65.7	21.2	11.1	0	0	2.0
Community	40.4	25.3	11.1	0	0	23.2

A key factor contributing to the positive perception of the VDP among patients was the relief of having an effective strategy for infant prophylaxis. Comments indicated that the VDP gave people hope and that patients were grateful and relieved to have something that could protect their babies. Other factors contributing to the positive perception of the program among patients were: awareness that the VDP brought about PMTCT, growing confidence in health workers, ease of administration, and availability of a take-home package. Government representatives appreciated the program as an innovative approach that made a difference in the health of their citizens, and respondents believe that this constituency perceived VDP as a mechanism for filling the gaps that limited the capacity of the MoH to propose or scale-up PMTCT programs.

Although positive perceptions of the VDP were lower among the community compared with the other constituencies, 66.0% of responses were still positive or very positive (Table 3). Qualitative analysis shows that the community appreciated the services that the VDP provided to mothers in the community and the community overall. The importance of education was also cited as a factor in the success of the program among the community and as a necessary element to address HIV-related stigma. Furthermore, the involvement of constituencies not typically involved in national heath programs was perceived to strengthen social support networks, which is believed to have contributed to a reduction in stigma against HIV-positive pregnant women.

## Discussion

This large international study demonstrates that the VDP acted as a catalyst for initiating PMTCT services, improving operational capacity and logistics of local institutions, and building partnerships that strengthen HIV/AIDS prevention and treatment strategies. More than half of respondents reported that the VDP helped to launch PMTCT services at the institutional level. These local programs established a foundation for more-widespread ARV therapy for PMTCT by training maternal and child health workers, educating and counseling women about HIV, and improving stock management system of ARVs. The availability of NVP that the VDP enabled created an environment that supported PMTCT uptake, as evidenced by the 85% of program mangers who reported that VDP improved access to PMTCT services. Improved access to PMTCT services is critical to a reduction in the number of HIV-positive children, as access to such programs correlates with sharp reductions in the number of newly infected children [17]. Geographic coverage of PMTCT services has expanded greatly in countries participating in the VDP, as demonstrated by the 78% of program managers who reported an increase in the number of PMCTCT facilities within their respective countries following the launch of the VDP. This improvement may also have broad beneficial effects because many countries have not scaled-up their PMCTC services despite an urgent need to do so [18]. As reported by half of the program managers, the VDP encouraged the development of national PMTCT policies [19]. These improvements may help to support improved PMTCT services, as capacity building and routine assessment of the quality of nursing care were reported to be key factors in the successful transition from single-dose NVP regimens to multi-dose ARV regimens [20]. These services enhance the health system by fostering better organization and integration of HIV prevention and HIV care services into the health care infrastructure.

In contrast to other reports [21], results from this impact study show that the VDP was associated with an increase in the number of antenatal care visits in half of the institutions and improvements in training capacity at 40% of institutions. This is a positive effect, especially given that staff shortages pose a significant challenge to scale-up of HIV care treatment services, including PMTCT, in resource-constrained countries [22]. Additionally, a study in 60 health facilities in Mainland Tanzania determined that average staff workload was below 100%, suggesting that increased training capacity might allow additional services to be provided without necessarily requiring additional hiring [16].

Results show that 79% of program managers reported that the VDP helped to reduce stigma against pregnant women who are HIV-positive. Qualitative analysis of questionnaire responses also identified stigma reduction as a key factor in the positive perception of the VDP among participating communities. This may help to further increase the number of pregnant women availing themselves of PMTCT services, as health worker stigma and discrimination against HIV/AIDS are factors that lead HIV-positive women to avoid delivering in health facilities [21].

The VDP may also provide longer-term benefits to participating institutions. 90% of respondents reported receiving outside funding to support PMTCT services and institutional relationships with these funding sources might facilitate future efforts to access funding and other health services. Institutions participating in the VDP, especially those that implemented the program in its first few years, were often first in their area to establish a base of PMTCT experience and expertise, which enabled them to apply successfully for further support from funding agencies. Implementation of the VDP also appears to have triggered changes at the national level that may have long-term positive effects both on PMTCT and the broader effort to prevent and manage HIV/AIDS [12].

Although the VDP was a donation program, it is important to note that many of the program’s positive effects resulted from the availability of NVP in the right place and at the right time rather than from the economic impact of receiving the drug free-of-charge. At the time that the VDP was launched, low-resource countries had an urgent need to adopt effective PMTCT strategies, and policy makers welcomed the program as a solution to a large and growing public health problem. As noted by several program managers, the VDP helped to initiate PMTCT services because it made an effective strategy available. A coordinated effort to identify HIV-positive pregnant women had not previously been undertaken because there was nothing to offer women who tested positive. The VDP was a valuable component of expanded HIV/AIDS care at a time when few health practitioners in the country had experience with PMTCT. For these countries and institutions, the availability of a new treatment option catalyzed systemic changes that are likely to have long-term effects on the HIV/AIDS epidemic and other aspects of health care. This underscores the need to continue to expand and/or establish programs that increase access to novel therapies.

It has been estimated that NVP represented only 1.2% of the value of the investment needed to establish PMTCT programs and that the bulk of funding for other aspects of these programs (training, antenatal and postnatal care, promotion and sensitization and bringing women into the health care system), was provided through public-private partnerships between Ministries of Health, NGOs and pharmaceutical companies that provide services in low and middle-income countries. This suggests that addressing public health issues will require cooperation among commercial, local, national, and international organizations. Such relationships will likely play a key role in improving PMTCT coverage in low- and middle-income countries, which has remained low [23].

Our study has several limitations. A key limitation of this study is that it represents the experiences and opinions of only 60% of all the institutions included in the VDP. We cannot determine if non-responding institutions have had similarly positive experiences with the program. There is a risk that institutions that were not satisfied with the VDP would not have invested the time to complete the online questionnaire. It should be noted, however, that a majority of non-responding institutions were approved for the VDP between 2001 and 2004 and were more likely to have already closed their VDP programs than institutions that had implemented the VDP more recently.

This study is also limited by the design approach, which measured the impact of the VDP in different areas at the program’s end. This approach does not allow us to compare the quality or type of PMTCT or other healthcare services provided before and after implementation of VDP, which could provide additional validation of the program’s impact on these services. However, the results of the survey utilized in this study appear to be robust and do provide important insight into the impact of a donation program on a variety of services and programs at the local, institutional, and national levels. Similarly, there are inherent limitations associated with data that are self-reported rather than obtained through objective assessment or observation. Additionally, the use of a standardized survey across different countries and types of institutions did not allow for some terms and categories to be defined with a high degree of specificity. For example, stigma associated with HIV/AIDS can be manifested in different ways depending on a patient’s local culture and societal norms. Thus, although 79% of program managers reported that the VDP helped to reduce stigma against HIV-positive pregnant women we are not able to provide greater detail as to how this reduction in stigma impacted these women’s social or economic status or their ability to access appropriate healthcare and HIV or pregnancy support services.

Another limitation is that 90% of respondents are African institutions, which may make it difficult to generalize the results to other resource-constrained regions. Additionally, the inclusion of 10 institutions outside of Africa results in a non-homogeneous sample population. The lower prevalence of HIV in non-African countries may lead to differences in attitudes and needs related to PMTCT and these differences could have impacted our results. However, a separate analysis of the African respondents alone showed similar results to those for the respondent population as a whole.

This study is also limited by the understanding of the specific role of the VDP in the context of multiple programs and funding initiatives in low-income countries. There was significant scale-up of international donor funding over the 11-year period of the VDP, especially from the United States Government (i.e. President’s Emergency Plan for AIDS Relief, PEPFAR), which makes it challenging to separate the impact of the VDP from the broader effects of multi-faceted initiatives like PEPFAR. Despite this limitation, results indicate that responding institutions perceived the VDP as a critical stepping-stone for building increased HIV capacity at both the institutional and national levels.

## Conclusion

In conclusion, our results demonstrate that private initiatives to increase access to novel health interventions can have a significant impact on public health issues and foster diverse partnerships among government agencies, commercial organizations, local institutions, and international NGOs. Because the scientific and public health communities were ripe to implement policy changes that increased the availability of a PMTCT intervention, the VDP was initiated at an appropriate time. This timing allowed the program to act as a trigger, or catalyst, for positive systemic changes at the institutional and national levels that helped to reduce vertical transmission of HIV. Importantly, these changes appear to have been triggered as a “bottom-up” response to a newly available intervention. This suggests that making an intervention available at the local level in resource-constrained countries can generate the needed momentum to encourage governments to adapt relevant policies in order to fully leverage new treatment options. It is important to note that new interventions should be introduced at a time when there is a consensus among the scientific and international communities that the intervention is appropriate and beneficial. More generally, our results highlight the potential and critical interest in promoting drug access to care program in HIV/AIDS and other public health domains, including non-communicable diseases such as cancer, diabetes, and cardiovascular diseases in low and middle-income countries. Expanding access to treatment will require input from multiple stakeholders, such as governments, NGOs, the scientific community, insurance companies, drugs companies, and others, to explore new alternatives than can include additional contributions to secure better coverage and patient outcomes. Organizations across the health care spectrum should consider and develop strategies that increase access to new interventions as part of a broader effort to address significant health issues.

## Competing interests

The authors declare no competing interests.

## Authors' contributions

JL conceived the study, wrote the manuscript and performed the analysis; MHB conceived the study, collected data and performed the analysis; MR conceived the study, collected data; KS performed the analysis; JS conceived the study and helped to draft the manuscript. All authors read and approved the final manuscript.

## Pre-publication history

The pre-publication history for this paper can be accessed here:

http://www.biomedcentral.com/1471-2458/13/470/prepub
